# Green Tea Catechins, (−)‐Catechin Gallate, and (−)‐Gallocatechin Gallate are Potent Inhibitors of ABA‐Induced Stomatal Closure

**DOI:** 10.1002/advs.202201403

**Published:** 2022-05-07

**Authors:** Kanane Sato, Shunya Saito, Kohsuke Endo, Masaru Kono, Taishin Kakei, Haruka Taketa, Megumi Kato, Shin Hamamoto, Matteo Grenzi, Alex Costa, Shintaro Munemasa, Yoshiyuki Murata, Yasuhiro Ishimaru, Nobuyuki Uozumi

**Affiliations:** ^1^ Department of Biomolecular Engineering Graduate School of Engineering Tohoku University Aobayama 6‐6‐07 Sendai 980‐8579 Japan; ^2^ Department of Biology Graduate School of Science University of Tokyo Bunkyo‐ku 113‐0033 Japan; ^3^ Department of Biosciences University of Milan Via G. Celoria 26 Milan 20133 Italy; ^4^ Institute of Biophysics National Research Council of Italy (CNR) Via G. Celoria 26 Milan 20133 Italy; ^5^ Graduate School of Environmental and Life Science Okayama University Tsushima Okayama 700‐8530 Japan

**Keywords:** calcium oscillation, catechin gallate, drought stress, green tea, stomata

## Abstract

Stomatal movement is indispensable for plant growth and survival in response to environmental stimuli. Cytosolic Ca^2+^ elevation plays a crucial role in ABA‐induced stomatal closure during drought stress; however, to what extent the Ca^2+^ movement across the plasma membrane from the apoplast to the cytosol contributes to this process still needs clarification. Here the authors identify (−)‐catechin gallate (CG) and (−)‐gallocatechin gallate (GCG), components of green tea, as inhibitors of voltage‐dependent K^+^ channels which regulate K^+^ fluxes in *Arabidopsis thaliana* guard cells. In *Arabidopsis* guard cells CG/GCG prevent ABA‐induced: i) membrane depolarization; ii) activation of Ca^2+^ permeable cation (*I*_Ca_) channels; and iii) cytosolic Ca^2+^ transients. In whole *Arabidopsis* plants co‐treatment with CG/GCG and ABA suppressed ABA‐induced stomatal closure and surface temperature increase. Similar to ABA, CG/GCG inhibited stomatal closure is elicited by the elicitor peptide, flg22 but has no impact on dark‐induced stomatal closure or light‐ and fusicoccin‐induced stomatal opening, suggesting that the inhibitory effect of CG/GCG is associated with Ca^2+^‐related signaling pathways. This study further supports the crucial role of *I*_Ca_ channels of the plasma membrane in ABA‐induced stomatal closure. Moreover, CG and GCG represent a new tool for the study of abiotic or biotic stress‐induced signal transduction pathways.

## Graphical abstract

Ingredients of green tea affect the growth of *Arabidopsis thaliana* under drought conditions. Catechin gallates and gallocatechin gallates inhibit plasma membrane‐located K^+^ channels and prevent Ca^2+^ entry in *Arabidopsis* guard cells. These catechin derivates are potent natural chemical regulators controlling plant growth and enabling the study of drought‐induced signaling pathways in guard cells.

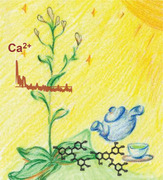

## 1 Introduction

When plants undergo environmental changes or biotic stress, they regulate the opening and closure of stomata to ensure transpiration, prevent water, loss or bacterial invasion to maintain intracellular homeostasis. ABA signal transduction plays a central role in this stomatal closure during drought stress.^[^ ^1^, ^2^, ^3^ ^]^ Drought stimulus elicits the synthesis of plant hormones such as ABA. This is followed by ABA binding to the ABA receptors in guard cell which initiates downstream responses including ion uptake and excretion, intracellular calcium ion (Ca^2+^) transient increase, generation of Reactive Oxygen Species, and protein phosphorylation/dephosphorylation, resulting in the excretion of ions, solutes, and water from guard cells which leads to a decrease in the turgor pressure and cell volume.^[^ ^4^, ^5^, ^6^, ^7^ ^]^

Plant cells contain potassium ions (K^+^) as the major cations for controlling membrane potential, cell volume changes, and enzymatic activity. Voltage‐dependent K^+^ channels which operate predominantly in the plasma membrane of guard cells^[^ ^3^, ^4^ ^]^ are well recognized for their role in promoting the accumulation and release of K^+^ which functions as an osmolyte for the fine tuning of the cell volume after intracellular Ca^2+^ increase. Cytosolic Ca^2+^ elevation is a major event in response to different stimuli in plant cells.^[^ ^8^ ^]^ On the other hand, information on the initiation mechanism of Ca^2+^ elevation is limited. Several reports indicate that the Ca^2+^ channel activity in guard cells is affected by changes in the plasma membrane potential.^[^ ^9^, ^10^ ^]^ In animal cells, it is well known that K^+^ flux across the membrane directly contributes to membrane potential regulation, and a set of Ca^2+^ channels and K^+^ channels cooperate in neurons and cardiac cells during Ca^2+^ entry and Ca^2+^ release, which also controls other K^+^ channels.^[^ ^11^ ^]^ Based on the situation in animals, cooperation between K^+^ channels and Ca^2+^ channels may also occur in signaling pathways in plant cells.

To dissect the physiological role of ion channels functioning in different signaling pathways, genetic analyses have been commonly pursued. However, since plants can compensate for the effects of genetic changes, the approach of genetic analysis using mutants is not always straightforward. In contrast, ion channel inhibitors can be used to rapidly block specific activities, thereby enabling the elucidation of signal transduction pathways more precisely and avoiding the problems resulting from genetic approaches. Chemical regulators used as medication to alleviate life‐threatening diseases need to be highly specific for their target molecules. For this reason, a large variety of animal ion channel inhibitors have been developed to treat arrhythmias, blood pressure abnormalities, and energy homeostasis disturbances.^[^ ^12^ ^]^ In plants, the blockage of ion channels by chemicals has not been studied in detail. Specific chemical regulators of ion transport activity would be potent modulators for the control of membrane potential, the activity of membrane transport and cell volume, consequently allowing plants to protect themselves against drought stress or other extreme environmental changes. Compounds that regulate ion channels on the cell surface may also have applications as efficient plant growth regulators and herbicides. To ensure the safety of such treatments and to prevent the persistence of chemicals in soil, biodegradable natural compounds instead of hazardous synthetic agrochemicals, have been considered for the development of nature‐friendly plant growth regulators.^[^ ^13^ ^]^

Green tea is a popular traditional beverage and its consumption around the globe amounts to ≈600 000 tons per year. Green tea has multimodal impacts on human health and has been shown to help alleviate or prevent various types of human disease.^[^ ^14^ ^]^ In addition to these effects in humans, active compounds contained in green tea waste suppress weed growth.^[^ ^15^ ^]^ Green tea is rich in catechins, a group of flavonoids distributed in tea plants, comprising 30–42% of the dry weight.^[^ ^16^ ^]^ The major catechins included in green tea leaves are (−)‐epicatechin (EC), (−)‐epigallocatechin (EGC), (−)‐epicatechin gallate (ECG), and (−)‐epigallocatechin gallate (EGCG), which account for 75% of tea polyphenols.^[^ ^17^ ^]^ The basic structure of catechins is comprised of three polyphenols, A‐ring, B‐ring, and C‐ring as their chemical backbone.^[^ ^16^ ^]^ In addition, the gallate derivatives contain a D‐ring, which is formed by esterification of gallic acid, as an auxiliary functional group on the flavonoid skeleton. Catechins have fundamentally water‐soluble features with multiple hydrophilic hydroxyl groups. Flavonoids have positive cardiovascular health and neuroprotective effects.^[^ ^18^ ^]^ EGCG, the most abundant catechin (59% of total catechins in green tea leaves), has anti‐infective effects^[^ ^19^ ^]^ and inhibits mammalian K^+^ channels.^[^ ^20^, ^21^, ^22^ ^]^ In accordance with this, catechin possesses phytotoxicity against plants including *Arabidopsis thaliana*.^[^ ^23^ ^]^ A mixture of green tea waste and rice bran suppressed weed population while enhancing spinach growth, showing its potential as a weed control without the use of hazardous synthetic agrochemicals.^[^ ^15^ ^]^

With this background information in mind, we decided to further examine the effects of green tea on plants. We observed that green tea exacerbated the reduction of growth caused by drought stress, without much effect on photosynthesis function in *A. thaliana*. To explore the molecular basis of this inhibitory effect, we examined the effect of a variety of catechins contained in green tea on the activity of ion channels in the plasma membrane of the guard cell. We identified (−)‐catechin gallate (CG) and (−)‐gallocatechin gallte (GCG) as inhibitors of K^+^ channels and *I*_Ca_ channels, which can rapidly abolish ABA‐induced Ca^2+^ elevation, disrupt membrane potential changes and prevent stomatal closure elicited by ABA. These findings support that plasma membrane *I*_Ca_ channels play a critical role in transient cytosolic Ca^2+^ increase for the initial steps of the drought‐induced signaling pathways in *A. thaliana*.

## 2 Results

### 2.1 Green Tea Inhibits *Arabidopsis* Growth during Drought Conditions

**Figure 1:**
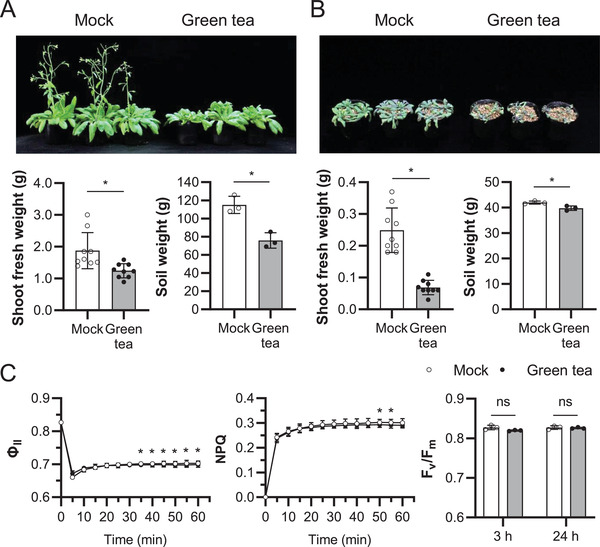
*Arabidopsis* responds to green tea. A,B) Representative *Arabidopsis* plants spray treated with green tea or water (top), fresh weight of the aerial parts of the plants (bottom left), and the weight of the soil after removal of aerial parts (bottom right). Plants were either grown under well‐watered conditions for the entire period (A) or watering was stopped after the spray treatment (B). Three‐week‐old plants were sprayed with green tea or water three times a week and photos were taken after approximately four weeks. Data corresponds to mean ± SD (*n* = 9 plants fresh weight, *n* = 3 weight of soil). Asterisks indicate significant difference between plants treated with water and that of green tea (* *p* < 0.05; two‐tailed Student's *t*‐test). C) Effects of 100% green tea on the photosynthetic activity. Green tea solution was sprayed on the adaxial side of the leaves. *Φ*_п_ was measured upon the transfer from the dark to light at 135 µmol m^−2^ s^−1^ (red and blue lights at 120 and 15 µmol m^−2^ s^−1^, respectively) for 60 min in the leaves after the 24 h (left panel). Non‐photochemical quenching (NPQ) was calculated as (*F*_m_ − *F*_m_′)/*F*_m_′ (center panel). *F*_v_/*F*_m_ of the leaves after 3 and 24 h of the treatments with water (−) or green tea (+) (right panel). Measurements were made after the 30 min dark treatment. Data shown corresponds to mean ± SD (*n* = 3). Asterisks indicate significant difference between plants treated with water and that of green tea at different time points (* *p* < 0.05, ns, not significant; two‐tailed Student's *t*‐test).

In addition to promoting human energy expenditure,^[^ ^24^ ^]^ green tea is known to impact plant growth.^[^ ^23^, ^25^, ^26^ ^]^ Therefore, we tested the effect of green tea on *Arabidopsis* plants (**Figure** **1**A,B). We used a commercial green tea product, “healthya” for which the composition of ingredients has been previously reported.^[^ ^24^ ^]^ The aerial parts (shoots) of the plants treated with green tea lost 34% of their fresh weight compared to untreated plants (Figure 1A). A reduction in weight of soil containing plant roots (34%) was also observed in treated plants compared to mock‐treated plants, which suggested that excess transpiration occurred from green tea‐treated plants. Reduced watering led to further decrease of the growth of plants treated with green tea (Figure 1B). Under these conditions, the fresh weight of plants treated with green tea was reduced by 72% with respect to that of control plants, however, the difference in reduction of soil weight was low (5.3%), probably due to the lesser overall soil moisture under reduced‐water conditions. On the other hand, spraying the adaxial side of leaves with green tea did not cause significant changes in PSII and PSI activities (Figure 1C and Figure S1, Supporting Information). These data suggested that some ingredients in green tea might adversely affect stomatal movements under drought conditions.

**Figure 2:**
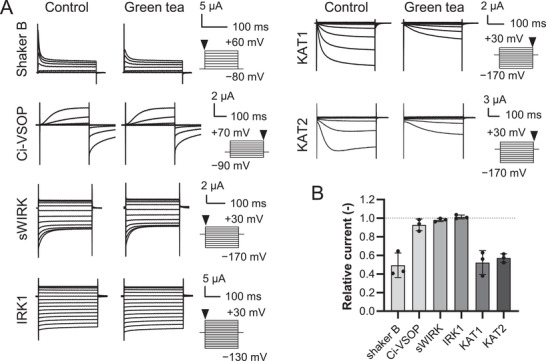
Inhibition of whole‐cell currents mediated by K^+^ channels by green tea. A) Representative current traces from *Xenopus* oocytes expressing Shaker B, Ci‐VSOP, sWIRK, IRK1, KAT1, and KAT2 with and without 5% green tea. B) Effect of green tea on Shaker B, Ci‐VSOP, sWIRK, IRK1, KAT1, and KAT2. Two‐electrode voltage clamp was carried out for each channel with or without 5% green tea in the external solution, and relative currents were plotted (*n* = 3, mean ± SD). The current value was collected at the end of the pulse at +70 mV for Ci‐VSOP and −170 mV for KAT1 and KAT2 or as instantaneous current value (5 ms after the initiation of voltage pulse) for Shaker B, sWIRK, and IRK1, at +60, −170, and −130 mV, respectively.

Different isoforms of voltage‐dependent potassium (K^+^) channels comprising the voltage sensor domain (S1‐S4) and the pore region (S5‐P‐S6) are expressed in guard cells where they play a key role in the regulation of stomatal movement (i.e., opening and closure).^[^ ^27^ ^]^ We speculated that water‐soluble compounds present in green tea might affect these plasma membrane‐located K^+^ channels. Catechin derivatives contained in green tea are known to regulate ion transport activities of *Shaker*‐type K^+^ channels^[^ ^20^, ^21^, ^22^ ^]^ by binding to the gap site between S4 and S5,^[^ ^28^ ^]^ and Ca^2+^ channels.^[^ ^29^, ^30^ ^]^ Prior to this test on plant channels, we tested whether green tea directly affects the activity of animal K^+^ channels using two‐electrode voltage clamp recordings of *Xenopus* oocytes (**Figure** **2**). Animal voltage‐dependent K^+^ channel, Shaker B showed a reduction in its instantaneous current when green tea was added (5% v/v)^[^ ^31^ ^]^ (Figure 2A,B). In contrast, different types of K^+^ channels consisting only of either the pore region, IRK1 (Kir2.1) and sWIRK, or of the voltage sensor domain forming the proton channel, Ci‐VSOP^[^ ^31^, ^32^, ^33^ ^]^ did not show significant inhibition by green tea. This suggested that catechins had some affinity to *Shaker*‐type ion channels, which was consistent with a previous report.^[^ ^28^ ^]^ We performed the same measurement on *Shaker*‐type K^+^ channels, KAT1 and KAT2, which function in the plasma membrane of *Arabidopsis* guard cells^[^ ^34^ ^]^ (Figure 2). The addition of green tea to the bath buffer decreased KAT1 and KAT2‐mediated currents by more than 40%, compared to the buffer without green tea (Figure 2A,B). These results indicated that some ingredients of green tea inhibited the activity of *Shaker*‐type K^+^ channels. According to the model of *Shaker*‐type K^+^ channels, KCNQ5 with ECG,^[^ ^28^ ^]^ catechins might be associated with S4‐S5 of *Arabidopsis Shaker*‐type ion channels.

### 2.2 Catechin Gallate (CG) and Gallocatechin Gallate (GCG) Inhibit the Activities of KAT1, KAT2, and GORK

**Figure 3:**
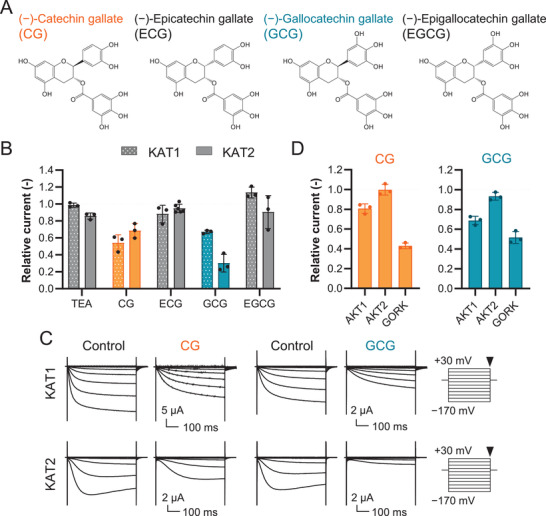
Inhibition of whole‐cell currents mediated by *Arabidopsis* K^+^ channels by CG and GCG. A) The chemical structures of the four catechins. B) Effect of four catechins on KAT1 and KAT2. Two‐electrode voltage clamp was carried out for each channel with or without 500 µm tetraethylammonium (TEA) or catechins in the external solution, respectively, and relative currents were plotted (*n* = 3–6, mean ± SD). The current value was collected at the end of the pulse −170 mV for KAT1 and KAT2. The data for TEA, CG, and GCG were the same as those depicted in Figure S3, Supporting Information. C) Representative current traces from *Xenopus* oocytes expressing KAT1 and KAT2 with and without 500 µm CG or GCG. D) Effect of CG and GCG on AKT1, AKT2, and GORK. Two‐electrode voltage clamp was carried out for each channel with or without 500 µm CG or GCG in the external solution, and relative currents were plotted (*n* = 3, mean ± SD). The current value was collected at the end of the pulse at −170 mV for AKT1, −155 mV for AKT2, and +50 mV for GORK.

To see if these soluble catechin derivates in green tea directly affect the activity of *Shaker*‐type K^+^ channels, we measured the effect of CG, ECG, GCG, EGCG, (+)‐catechin (C), EC, (−)‐gallocatechin (GC), andEGC on K^+^ channels expressed in *Xenopus* oocytes (**Figure** **3**A–C and Figure S2, Supporting Information). Both CG and GCG inhibited KAT1 and KAT2 (Figure 3A,B). Remarkably, ECG and EGCG, which are diastereomer of CG and GCG, were less effective in reducing the current amplitudes of KAT1 and KAT2. C, EC, GC, and EGC did not affect the current amplitude significantly (Figure S2, Supporting Information). The specificity of KAT1 and KAT2 inhibition by CG and GCG was investigated further with a commonly used K^+^ channel inhibitor, tetraethylammonium (TEA). TEA is a well‐known inhibitor of KAT1 activity as demonstrated by oocyte recordings.^[^ ^35^ ^]^ The dose‐dependent inhibition of KAT1 and KAT2 by TEA, CG, and GCG is shown in Figure S3, Supporting Information. TEA (0.5 mm) inhibited KAT1 by 1% and KAT2 by 14%, which was much less than the inhibition achieved by 0.5 mm CG and GCG (Figure 3B and Figure S3, Supporting Information). We next tested the effect of CG and GCG on AKT1, AKT2 and GORK, which are also present in the plasma membrane of *Arabidopsis* guard cells^[^ ^34^ ^]^ (Figure 3D). GORK was strongly inhibited by CG and GCG, whereas AKT1 and AKT2 were less sensitive to CG and GCG. These data showed that CG and GCG have stronger inhibitory effects on KAT1, KAT2, and GORK than TEA.

### 2.3 Effect of CG and GCG on Stomatal Closure

**Figure 4:**
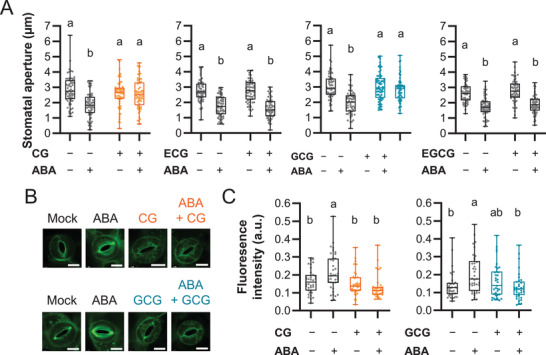
Inhibition of ABA‐induced stomatal closure by CG and GCG. A) Stomatal aperture of leaf epidermal strips treated with CG, ECG, GCG, or EGCG. The epidermal strips were incubated in closing buffer for 2 h before addition of 500 µm CG, ECG, GCG, or EGCG and 10 µm ABA and incubation for 2 h more. +, added; −, no addition. The data are plotted as boxplots (median and interquartile) and whiskers correspond to minimum and maximum values (*n* = 60). Bars marked with different letters are significantly different (*p* < 0.05) by one‐way ANOVA with Tukey–Kramer test. B,C) Inhibition of plasma membrane potential depolarization by CG/GCG in ABA‐treated guard cells. Representative image of guard cells treated with water, 500 µm CG or GCG with or without 10 µm ABA (B). Higher fluorescence of DiBAC_4_(3) indicates plasma membrane depolarization (C) (*n* = ≈35–39). Scale bar represents 10 µm. All data are plotted as boxplots (median and interquartile) and whiskers correspond to minimum and maximum values. Bars marked with different letters are significantly different (*p* < 0.05) by one‐way ANOVA with Tukey–Kramer test.

The increased sensitivity of plants treated with green tea to drought stress (Figure 1) and the impaired functionality of guard cell‐expressed K^+^ channels achieved by CG/GCG application (Figure 3) allowed us to hypothesize that the catechins might affect stomatal closure induced by the stress hormone ABA which is synthesized in response to drought stress.^[^ ^3^ ^]^ To evaluate the effect of catechins on ABA‐induced stomatal closure, pre‐opened stomata were treated with ABA alone or in combination with CG/GCG or their diastereomer ECG/EGCG (**Figure** **4**). Application of CG and GCG on guard cells prevented ABA‐induced stomatal closure (Figure 4A). In contrast, the diastereomer, ECG and EGCG, had no effect, which confirmed that the effect of CG and GCG on guard cell function was specific. Based on the data in Figure 4A, we predicted that other responses in the ABA‐induced stomatal closure process might also be affected by CG/GCG application. We examined the effect on the plasma membrane potential of ABA‐treated guard cells using a translational membrane potential dye (DiBAC_4_(3))^[^ ^36^ ^]^ (Figure 4B,C). The increase in fluorescence after ABA treatment of pre‐opened stomata, indicating depolarization of the membrane potential, was absent in CG or GCG‐treated cells. It was thus shown that adding CG or GCG together with ABA prevents the depolarization occurring in ABA‐treated cells, which might be the cause of stomatal closure inhibition.

### 2.4 CG/GCG Attenuated Cytosolic Ca^2+^ Oscillations in Guard Cells

**Figure 5:**
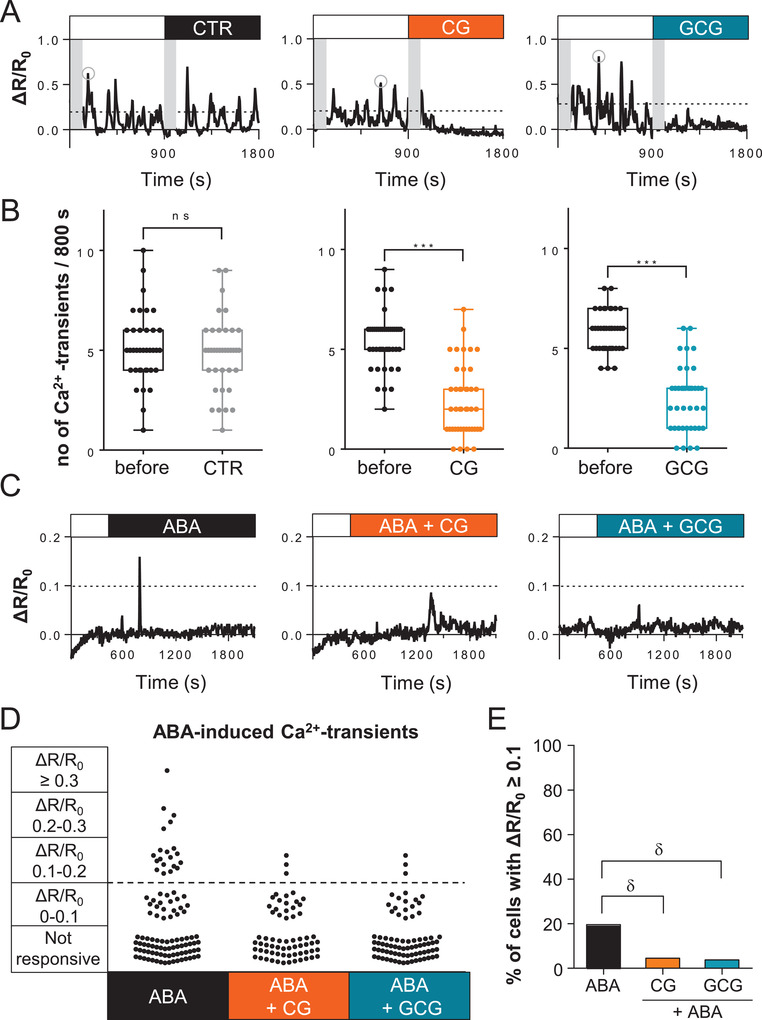
CG and GCG suppressed cytosolic Ca^2+^ oscillations in guard cells. A,B) Normalized cpVenus/CFP ratios, as Δ*R*/*R*_0_ of single representative guard cells expressing the NES‐YC3.6 Cameleon Ca^2+^ indicator, treated at 900 s with the closing buffer (CTR), CG, or GCG. The grey areas represent those time windows at the beginning of the image acquisition and just after addition of CG, GCG, or closing buffer, that were not considered for the determination of the number of Ca^2+^ transients. Dashed lines represent the threshold (30% of the highest value) above which peaks were counted as Ca^2+^ transients during data acquisition (bottom). All data are plotted as boxplots and whiskers indicate minimum and maximum values. The line in the middle of the box corresponds to the average. Error bars represent ± SD (*n* = 5 using 38 guard cells for the control, 43 guard cells for CG, and 39 guard cells for GCG). *** *p* < 0.005, ns, not significant (two‐tailed Student's *t*‐test). C) Normalized cpVenus/CFP ratios, as Δ*R*/*R*_0_ of single representative guard cells expressing the NES‐YC3.6 Cameleon Ca^2+^ indicator, treated at 600 s with 10 µm ABA, 10 µm ABA + 500 µm CG, and 10 µm ABA + 500 µm GCG. Dashed lines represent the threshold above which we considered the peaks as bona fide ABA‐induced cytosolic Ca^2+^ transients. D) Scatter plots reporting all measured transients for each condition tested. E) Percent (%) of cells showing transients with a Δ*R*/*R*_0_ ≥ 0.1 (dashed line in (D)). The total number of analyzed guard cells for each treatment were 102 for ABA alone (*n* = 7), 66 for ABA + CG (*n* = 5), and 70 for ABA + GCG (*n* = 5). *δ p* < 0.01 (*χ* ^2^ test).

Dysregulation of the plasma membrane potential due to CG/GCG may disturb the activity of Ca^2+^ permeable voltage‐dependent channels in the plasma membrane. We assessed the impact of CG/GCG on spontaneous cytosolic Ca^2+^ oscillations in guard cells, commonly observed in freshly prepared *Arabidopsis* epidermal strips.^[^ ^37^ ^]^ *Arabidopsis* plants expressing the NES‐YC3.6 Cameleon Ca^2+^ indicator were used to monitor in vivo cytosolic Ca^2+^ dynamics of guard cells before and after the addition of CG, or GCG in independent experiments^[^ ^38^ ^]^ (**Figure** **5**A,B). Both CG and GCG treatments strongly and immediately reduced the number of spontaneous Ca^2+^ oscillations during the observed time window. In guard cells, Ca^2+^ signaling is important for the regulation of stomatal movements^[^ ^39^, ^40^ ^]^ and specifically it is known to enhance the efficiency of ABA‐induced stomatal closure during drought stress.^[^ ^6^ ^]^ Indeed, ABA induces cytosolic Ca^2+^ elevation in guard cells when added to pre‐opened stomata.^[^ ^5^, ^6^, ^41^ ^]^ We therefore tested the effect of CG and GCG on ABA‐induced cytosolic Ca^2+^ transients. As previously reported, the guard cells of open stomata do not show the spontaneous cytosolic Ca^2+^ transients observed in freshly prepared epidermal strips.^[^ ^42^, ^43^, ^44^ ^]^ Administration of ABA induced cytosolic Ca^2+^ transients which were mostly absent when CG or GCG were added together with ABA (Figure 5C). Specifically, while ABA induced cytosolic Ca^2+^ transients of different magnitudes (Figure 5D), the presence of CG and GCG prevented any transients with values of a normalized cpVenus/CFP ratio (Δ*R*/*R*_0_) above 0.2 (Figure 5C). Even when we considered cells with small cytosolic Ca^2+^ increases (0.1–0.2), the number of responding cells was dramatically reduced under the presence of CG or GCG (Figure 5E).

### 2.5 CG/GCG Blocked ABA‐Induced *I*_Ca_ Channels in the Plasma Membrane of Guard Cells Protoplasts

**Figure 6:**
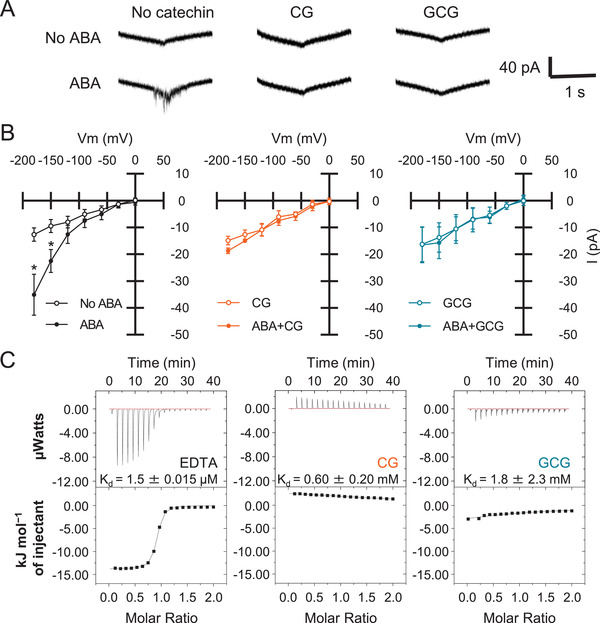
Patch clamp recoding of *I*_Ca_ channels inhibited by CG/GCG. A) Representative *I*_Ca_ current traces of guard cell protoplasts are shown. Ramp voltage pulses were elicited from +20 to −180 mV with −200 mV s^−1^. B) Current–voltage relationships of *I*_Ca_ currents without catechin (left) (*n* = 6, *p* < 0.05 at −180 mV, no ABA versus 10 µm ABA) or with 500 µm CG (middle) (*n* = 3, *p* = 0.28 at −180 mV, no ABA versus 10 µm ABA) or 500 µm GCG (right) (*n* = 3, *p* = 0.52 at −180 mV, no ABA versus 10 µm ABA) are shown. Error bars represent SEM. Asterisks indicate significant difference between no ABA and 10 µm ABA at different time points (* *p* < 0.05; two‐tailed Student's *t*‐test). C) Evaluation of binding of Ca^2+^ to CG and GCG. ITC data was obtained by titrating varying concentrations of EDTA, CG, and GCG to 4 mm CaCl_2_. The lower panel represents the fitted curve of integrated peak area in the upper panel versus the molar ration of Ca^2+^.

ABA induces guard cell Ca^2+^ elevation at least partially via activation of the plasma membrane hyperpolarization‐activated Ca^2+^‐permeable cation (*I*_Ca_) channels.^[^ ^3^, ^42^, ^45^, ^46^, ^47^ ^]^ To evaluate the effect of CG and GCG against *I*_Ca_ channels, we performed whole‐cell patch clamp recordings in guard cell protoplasts (**Figure** **6**A,B). Typical *I*_Ca_ currents were observed in guard cell protoplasts treated with ABA. The ABA‐activated *I*_Ca_ current was clearly suppressed by CG and GCG. Overall, these experiments clearly showed that CG and GCG prevented ABA‐induced Ca^2+^ entry into guard cells. To rule out that the lack of *I*_Ca_ current was due to a direct chelation of Ca^2+^ by CG or GCG, ITC measurements were performed (Figure 6C). Both CG and GCG displayed weaker binding ability of Ca^2+^ compared to the positive control of EDTA. Note that we used EDTA but not EGTA as control since we predicted that CG/GCG would have a relatively low affinity for Ca^2+^. These results indicate that, in vivo, CG and GCG dampened cytosolic Ca^2+^ oscillations by inhibiting plasma membrane *I*_Ca_, leading us to hypothesize that CG and GCG may have an impact on Ca^2+^ signaling.

### 2.6 CG and GCG Inhibited ABA‐Induced Stomatal Closure in Whole Plants

**Figure 7:**
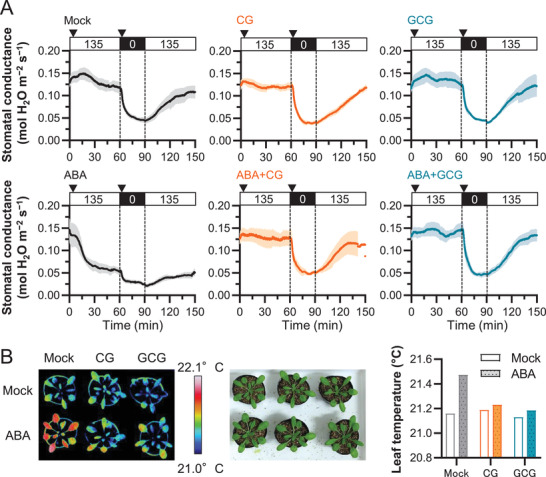
Whole‐plant recordings of changes in stomatal conductance and leaf temperature in response to ABA with or without CG and GCG. A) Dynamics of stomatal conductance in *Arabidopsis* leaves. Stomatal conductance in response to treatment with ABA or CG or GCG with/without ABA was monitored in the light and in the dark (mean ± SEM, Mock: 0.0 ≤ time [*t*, min] ≤ 120.0, *n* = 6; 120.0 < *t* ≤ 150.0, *n* = 5, CG: 0.0 ≤ *t* ≤ 150.0, *n* = 4, GCG: 0.0 ≤ *t* ≤ 127.8, *n* = 4; 127.8 < *t* ≤ 150.0, *n* = 3, ABA: 0.0 ≤ *t* ≤ 150.0, *n* = 4, ABA + CG: 0.0 ≤ *t* ≤ 60.0, *n* = 4; 60.0 < *t* ≤ 141.0, *n* = 3; 141.0 < *t* ≤ 150.0, *n* = 2, ABA + GCG: 0.0 ≤ *t* ≤ 150.0, *n* = 4). Triangles indicate application of the corresponding treatment at the start of the experiment and after transition to the dark. B) Representative false‐color infrared image of plants treated with CG or GCG. The 3‐4‐week‐old plants were sprayed with 500 µm CG or 500 µm GCG without or with 10 µm ABA. The infrared thermography was taken 3 h after the treatment. Quantified differences of leaf surface temperature are shown for a representative experiment. Similar results were obtained in three independent experiments.

To corroborate the CG/GCG impact on ABA‐induced stomatal closure, in vivo responses of intact guard cells were evaluated by monitoring stomatal conductance with a gas exchange system (**Figure** **7**A). Responses after addition of CG or GCG alone were similar to the mock treatment. Addition of ABA rapidly induced stomatal closing in the light within 60 min (bottom left panel in Figure 7A). However, when CG or GCG was applied in combination with ABA, ABA‐induced stomatal closing was prevented (bottom center and right panels in Figure 7A), while a light‐induced opening was not inhibited. These data indicate that CG/GCG acted as inhibitors of ABA‐induced stomatal closure. A long‐term observation of CG/GCG‐treated plants was also performed (Figure 7B). Thermal imaging of plants treated with CG/GCG did not reveal any differences in their leaf temperature compared to non‐treated plants, indicating no change in water evaporation. Plants treated with ABA alone showed an increase in leaf temperatures which was inhibited by the addition of CG/GCG. This suggested that CG/GCG hampered ABA‐induced stomatal closure.

### 2.7 Involvement of K^+^ Channels in Pathogen‐Induced Stomatal Closure

**Figure 8:**
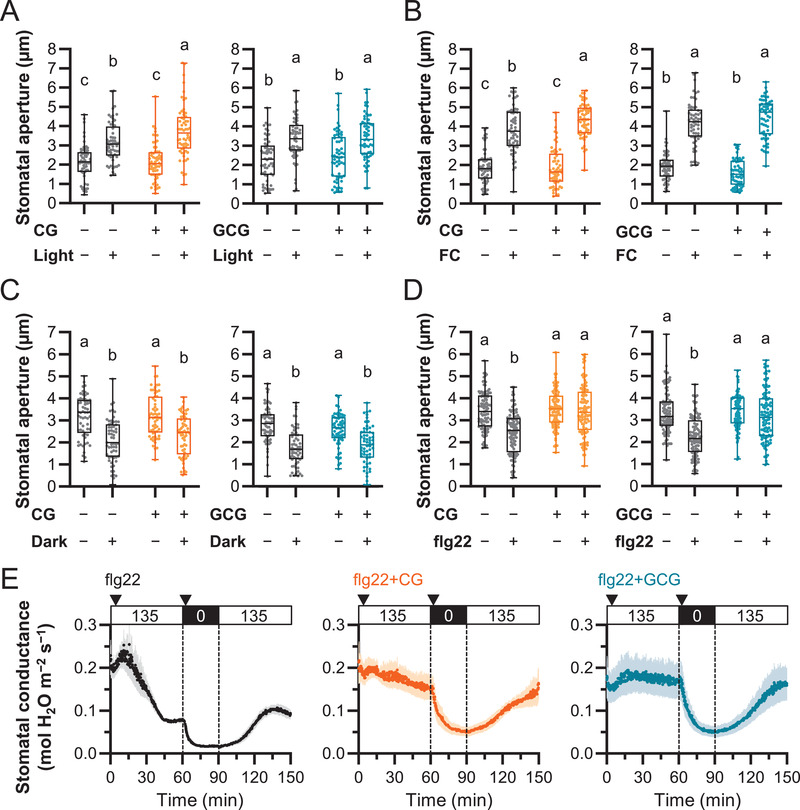
Effect of CG/GCG on stomatal aperture during exposure to different stimulants. A) Effect of CG or GCG on light‐induced stomatal opening. Leaf epidermal strips were incubated in opening buffer without or with 500 µm CG or GCG for 3 h in the light or in the dark (*n* = 60). +, added; −, no addition. B) Effect of CG and GCG induction of stomatal opening by fusicoccin (FC). Leaf epidermal strips were incubated in opening buffer without or with 500 µm CG, 500 µm GCG, or 10 µm FC for 3 h (*n* = 60). +, added; −, no addition. C) Effect of treatment with CG or GCG on dark‐induced stomatal closing (*n* = 60). Epidermal strips were incubated in closing buffer for 2 h in the dark, then 500 µm CG or GCG were added and strips were incubated for additional 2 h under in the dark. +, added; −, no addition. D) Effect of treatment with CG or GCG on flg22‐induced stomatal closing. Epidermal strips were incubated in closing buffer for 2 h and 500 µm CG or GCG or 5 µm flg22 were added and strips were incubated for 2 h more in the light (*n* = 100). +, added; −, no addition. E) Stomatal conductance in *Arabidopsis* leaves during treatment with flg22 in the absence or presence of CG or GCG (mean ± SEM, flg22: 0.0 ≤ time (*t*, min) ≤ 150.0, *n* = 8, flg22 + CG: 0.0 ≤ *t* ≤ 149.2, *n* = 5; 149.2 < *t* ≤ 150.0, *n* = 4, flg22 + GCG: 0.0 ≤ *t* ≤ 150.0, *n* = 7). Treatments were reapplied (20 µL) 5–10 min after the transition from light to dark. Data in panel (A–D) are shown as boxplots (median and interquartile) and whiskers correspond to minimum and maximum values. Bars marked with different letters are significantly different (*p* < 0.05) by one‐way ANOVA with Tukey–Kramer test.

The data in Figures 4, 5, 6, 7 suggested that CG/GCG affected the membrane potential regulation by KAT1, KAT2, or GORK as well as Ca^2+^ influx and Ca^2+^ transients in guard cells. To test the effect of CG/GCG on stomatal movement induced by other cues, we applied various stimuli to *Arabidopsis* leaf epidermal strips, with or without the presence of CG/GCG (**Figure** **8**). CG/GCG did not affect light‐induced stomatal opening, which was consistent with the data in Figure 7A and stomatal opening mediated by fusicoccin, a potent activator of H^+^‐ATPase^[^ ^48^ ^]^ (Figure 8A,B). CG/GCG did not prevent dark‐induced stomatal closure (Figure 8C). However, stomatal closure induced by the flagellin peptide, flg22, was impaired by the application of CG or GCG (Figure 8D).^[^ ^49^ ^]^ Further in vivo assays of stomatal conductance supported the data in Figure 8D: flg22 alone elicited rapid reduction of stomatal conductance in the light, which was not observed when CG/GCG together with flg22 was applied (Figure 8E). This suggested that flg22‐induced stomatal closure pathway might contain CG/GCG‐sensitive ion channels.

## 3 Discussion

This study demonstrates that CG and GCG, natural compounds in green tea, were effective tools for dissection of the role of ion channels located in the plasma membrane in governing guard cell Ca^2+^ oscillation in the signal pathway of ABA‐induced guard cell closure. The strong inhibition of plasma membrane Ca^2+^ influx and suppression of Ca^2+^ oscillations by CG and GCG indicated that *I*_Ca_ channels in the plasma membrane play an important role in cytosolic Ca^2+^ elevation in ABA signaling pathways (Figure 5, 6, 7). The use of pharmacological agents to resolve biological functions and phenotypes is always accompanied by problematic side effects. Hence, it is challenging to identify useful regulators for this purpose, and limited progress has been made in the dissection of the molecular mechanism of ion channels by using chemical regulators. We cannot rule out the possibility that CG/GCG might have other targets in the ABA signaling pathway. On the other hand, CG/GCG reduced the activity of KAT1, KAT2, and GORK (Figure 3) and strongly inhibited *I*_Ca_ currents (Figure 6), which likely caused the perturbation of ABA‐induced stomatal closure (Figures 4 and 7). Despite their potential shortcomings, CG/GCG were useful in elucidating short‐term molecular mechanisms which are challenging to investigate by genetic methods alone. Although ion channel specific inhibitors without side effects still remain to be identified, CG/GCG were effective molecules in uncovering the crucial involvement of ABA‐induction of plasma membrane Ca^2+^ entry channels.

There have been numerous reports on the physiological function of catechins in humans and most reports have focused on the major catechin in green tea, EGCG.^[^ ^50^ ^]^ Molecular level analyses revealed that EGCG affects electrophysiological features such as cardiac repolarization and electrical excitability of neurons, controlling rat Kv1.5,^[^ ^20^ ^]^ Kir6.2,^[^ ^21^ ^]^ and cardiac hERG,^[^ ^22^ ^]^ several Ca^2+^ channel/receptors in prostate cancer cells^[^ ^29^ ^]^ and human Cav3.1 and Cav3.2.^[^ ^30^ ^]^ KCNQ5 is also activated by EGC as well as EGCG, leading to the conclusion that EGC interacts with the connecting sites between the voltage sensor domain of S4 and S5.^[^ ^28^ ^]^ Moreover, this position serves as binding sites of other small chemicals, that is*, γ*‐aminobutyric acid (GABA).^[^ ^51^ ^]^ Analogous catechin derivatives, CG and GCG are classified as minor catechins in green tea, and their physiological impact on animals and plants is unclear. This study identified CG and GCG as inhibitors of *Arabidopsis* KAT1 and KAT2 with a higher efficacy than a general K^+^ channel inhibitor, TEA, while their diastereomer, ECG and EGCG did not have such effects (Figure 3). CG/GCG strongly inhibited *I*_Ca_ and the distinct chirality between CG/GCG and ECG/EGCG was evident in their different effect on ABA‐induced stomatal closure (Figure 4A).

The ABA signaling pathway is a complex network of coexisting and connected Ca^2+^‐dependent and Ca^2+^‐independent pathways.^[^ ^6^, ^52^, ^53^ ^]^ When plant cells are subjected to drought stress, binding of ABA to ABA receptors, PYR/PYL/RCAR triggers activation of Ca^2+^‐independent OST1/SnRK2.6 kinase, leading to activation of SLAC anion channels via phosphorylation and concomitant K^+^ release from the cells by GORK.^[^ ^3^, ^54^ ^]^ On the other hand, it is known that stomatal closure is initiated by Ca^2+^ entry into the guard cells through ABA‐activated Ca^2+^ channels in response to hyperpolarization of the plasma membrane.^[^ ^42^, ^45^, ^46^, ^55^, ^56^ ^]^ This is followed by Ca^2+^‐induced Ca^2+^ release from endomembrane stores (CICR) and activation of effector proteins by Ca^2+^‐binding proteins.^[^ ^5^, ^9^, ^57^, ^58^, ^59^ ^]^ Both pathways result in the activation of SLAC1 for completion of stomatal closure. The order of the Ca^2+^‐dependent and Ca^2+^‐independent pathways has not yet been established but several suggestions have been made.^[^ ^3^, ^5^ ^]^ The Ca^2+^‐independent signaling process involving OST1/SnRK2.6 is followed by a long‐term response involving a Ca^2+^‐dependent signaling process which includes gene expression.^[^ ^60^ ^]^ The most accepted concept is that Ca^2+^ elevation amplifies the ABA signaling pathway in guard cell.^[^ ^3^, ^54^ ^]^ If this process is applied to our results, CG/GCG might interrupt the signal flow following the Ca^2+^ independent pathway.

This study showed that the addition of CG/GCG induced a significant reduction of spontaneous cytosolic Ca^2+^ oscillation and abrogation of *I*_Ca_ channel activity in the plasma membrane of guard cells (Figure 5). It is unclear whether CG/GCG directly inhibited *I*_Ca_ channels or whether interference with K^+^ channels by CG/GCG adversely affected *I*_Ca_ channels (Figures 3 and 6). Ca^2+^ channels responsible for *I*_Ca_ in ABA signaling remain to be identified. In pathogen‐associated molecular patterns (PAMPs), the plasma membrane‐located Ca^2+^ permeable channels, CNGC2/4 and OSCA1.3/1.7 mediate Ca^2+^ entry from the extracellular space.^[^ ^61^, ^62^ ^]^ Given that CG/GCG mainly affected *Shaker*‐type ion channels, CNGC might be a possible target channel for CG/GCG binding in the ABA‐signaling pathway. In animal cells, K^+^ channels are involved in the generation of membrane potential oscillation^[^ ^63^, ^64^, ^65^ ^]^ and intracellular Ca^2+^ oscillation.^[^ ^66^ ^]^ Opening of K^+^ channel, Kv1.3 elicits membrane depolarization, and then to opening of a Ca^2+^ channel, IKCa1 causes cytosolic Ca^2+^ elevation.^[^ ^67^ ^]^ If a similar cooperation between K^+^ channels and Ca^2+^ channels should occur in plants, CG/GCG‐induced disturbance of proper function of hyperpolarization‐activated K^+^ channels (KAT1/KAT2) and depolarization‐activated K^+^ channels (GORK) might result in attenuation of cytosolic Ca^2+^ oscillation mediated by yet unidentified *I*_Ca_ channels in the plasma membrane.^[^ ^9^, ^10^ ^]^ The driving force for activation of Ca^2+^ channels in the plasma membrane during ABA‐induced stomatal closure remains unknown.^[^ ^10^, ^42^ ^]^ Further studies, such as identification of *I*_Ca_ channels are necessary to clarify the assumption of a connection between K^+^ channels and Ca^2+^ channels in the plasma membrane.

Voltage‐dependent ion channels contribute to the sensing and regulation of membrane potential changes in neurons and cardiac cells. Similarly, plant homologous voltage‐dependent K^+^ channels, KAT1/2 and GORK, likely participate in the ABA signal transduction pathway not only by supply or removal of K^+^ into/from the cells, but also by participating in modulating the activities of other channels/transporters including Ca^2+^ channels. As inhibitors of KAT1, KAT2, and GORK, CG and GCG could be potent chemical tools for the dissection of the initial events in the drought‐induced and pathogen‐induced signaling pathways. This study provides the first evidence that CG and GCG, natural compounds in green tea, may open up new ways for uncovering the role of plasma membrane‐located K^+^ channels and ABA‐induced Ca^2+^ elevation in the initial stages of guard cell shrinkage. For agricultural applications, CG and GCG have potential as chemical regulators of plant growth or as natural herbicides, making use of green tea waste produced in the beverage industry.

## 4 Experimental Section

### Recordings in *Xenopus laevis* Oocytes

For expression in *Xenopus laevis* oocytes the cDNA encoding KAT1, KAT2, AKT1, AKT2, GORK, CBL1, and CIPK23 were subcloned into the multicloning sites of a pYES2‐derived vector (Invitrogen, Carlsbad, CA, USA).^[^ ^68^ ^]^ cRNAs were synthesized from *Not*I‐linearized plasmids using an in vitro transcription kit (Ambion, Austin, TX, USA). Oocytes were microinjected with 10 ng of cRNA in 50 nL per oocyte and then incubated for a 1–3 day in Barth's buffer containing 88 mm NaCl, 1 mm KCl, 0.41 mm CaCl_2_, 0.33 mm Ca(NO_3_)_2_, 1 mm MgSO_4_, 2.4 mm NaHCO_3_, 5 mm HEPES‐NaOH, and 50 mg L^−1^ gentamicin sulfate (pH 7.3) at 18 °C. During two‐electrode voltage clamp recordings for KAT1, KAT2, AKT1, and AKT2, oocytes were perfused with a solution composed of 120 mm KCl, 1 mm MgCl_2_, and 1 mm CaCl_2_ 10 mm HEPES‐NaOH (pH 7.3). For the recordings of SKOR and GORK, 12 mm KCl and 108 mm NaCl were added instead of 120 mm KCl. Bath solutions for the remaining transporters were as follows: for IRK1, 2 mm NaCl, 96 mm KCl, 1 mm MgCl_2_, 1.8 mm CaCl_2_, and 1 mm HEPES‐KOH (pH.7.4),^[^ ^32^ ^]^ for salmon weakly IRK (sWIRK), 90 mm KCl, 3 mm MgCl_2_, and 1 mm HEPES‐KOH (pH.7.4),^[^ ^69^ ^]^ for drosophila Shaker B, 140 mm NaCl, 2 mm KCl, 2 mm MgCl_2_, and 10 mm HEPES‐NaOH (pH.7.2),^[^ ^31^ ^]^ for mouse voltage‐sensor domain‐only protein VSOP, 88 mm NaCl, 1 mm KCl, 1 mm MgCl_2_, 1 mm CaCl_2_, and 100 mm HEPES‐NaOH (pH.7.4).^[^ ^70^ ^]^ Voltage clamp protocols used were as follows: −80 mV to +60 mV, +20 mV increments, 300 ms test pulse with holding potential of −80 mV for Shaker B, −90 mV to +70 mV, +20 mV increments, 500 ms test pulse with holding potential of −40 mV for Ci‐VSOP, −130 mV to +30 mV, +10 mV increments, 500 ms test pulse with holding potential of −40 mV for IRK1 and −170 mV to +30 mV, +20 mV increments, and 500 ms test pulse with holding potential of −40 mV for sWIRK. Protocols for *Arabidopsis* K ^+^ channels are described elsewhere.^[^ ^71^, ^72^, ^73^, ^74^ ^]^ Recordings and data analysis were performed using an AxoClamp 2B amplifier (Axon Instruments) and an Axon Digidata 1550 System (Molecular Devices).

### Patch Clamp Recordings on Guard Cell Protoplasts

Guard cell protoplasts were prepared from *Arabidopsis* rosette leaves as described previously.^[^ ^4^ ^]^ The pipette solution for *I*_Ca_ current recording contained 10 mm BaCl_2_, 4 mm EGTA, and 10 mm HEPES‐Tris (pH 7.1), which was adjusted to 500 mOsm with D‐sorbitol. 5 mm NADPH and 0.1 mm DTT were freshly added to the pipette solution. The bath solution contained 100 mm BaCl_2_, and 10 mm MES‐Tris (pH 5.6) which was adjusted to 485 mOsm with D‐sorbitol. For catechin treatment, 500 µm GC or GCG was freshly added to the bath solution. Whole‐cell *I*_Ca_, currents were recorded using a CEZ‐2200 patch‐clamp amplifier (NIHON KOHDEN, Tokyo, Japan) and pCLAMP 8.1 software (Molecular Devices, Inc., CA, USA). Ramp voltage pulses were elicited from +20 to −180 mV with −200 mV s^−1^ from 0 mV as a holding potential. Control whole‐cell currents were recorded 10 times with a 10‐s interval. After control current recording, ABA was added to the bath solution and then ABA‐activated *I*_Ca_ currents were recorded 10 times with a 10‐s interval. The average current obtained from the 10 traces was used for current–voltage relationships. Liquid junction potential was not corrected and leak currents were not subtracted.

### Growth Test of *Arabidopsis* Plants and Stomatal Aperture Measurements

The green tea used in this study was a commercial prepared beverage, healthya (Kao Co., Tokyo, Japan).^[^ ^24^ ^]^ C, GC, CG, GCG, EC, EGC, ECG, and EGCG (Nagara Science Co. Gifu, Japan) were used in this study. *A. thaliana* (Col‐0) were grown in soil in a growth chamber with 16 h light (80 µmol m^−2^ s^−1^) and 8 h dark at 20 °C.^[^ ^75^ ^]^ To test the effect of green tea on plant growth, green tea or water were sprayed onto the leaves of three‐week‐old *Arabidopsis* plants. For measuring stomata aperture, excised rosette leaves from 3‐4‐week‐old plants were floated on opening buffer (30 mm KCl, 10mm MES‐KOH pH 6.0) overnight in the dark. Leaves were then chopped in a blender (700BUJ WARING) for 30 s, and the resulting material was filtered through a 100‐µm nylon mesh. The epidermis left on the mesh was incubated in the same buffer with 0.2% DMSO added and supplemented with 500 µm CG, 500 µm GCG (Funakoshi Co., Ltd.), or 10 µm fusicoccin (FC, Enzo Life Sciences, Inc.) for 3 h in light. The width of the stomata was measured under a bright field microscope. To measure stomatal movement induced by ABA or darkness, the epidermis fragments isolated as described above were incubated in closing buffer (5 mm KCl, 50 µm CaCl_2_, 10 mm Tris‐MES [pH 6.15]) for 2 h in the light. This was followed by 2 h treatments with 500 µm CG, 500 µm GCG, 10 µm ABA, or 5 µm flg22 (kindly provided by Takashi Shiina) in the dark. For false‐color infrared imaging of plants, 3‐4‐week‐old plants were sprayed with water containing 0.1% DMSO and 0.02% Silwet L‐77, 500 µm CG, 500 µm GCG, or 10 µm ABA in water containing 0.1% DMSO and 0.02% Silwet L‐77 and after 3 h the surface temperature was determined with an InfReC R550 camera (Nippon Avionics, Japan). Image analysis was performed using InfReC Analyzer NS9500 standard software (Nippon Avionics, Japan).

### Isothermal Titration Calorimetry (ITC) Measurements

ITC was performed with a MicroCal iTC200 microcalorimeter (Malvern Panalytical, Netherland). For the measurement, 4 mm CG, GCG, or EDTA was added to 0.4 mm CaCl_2_ in 10 mm MES‐NaOH pH 6.0 at 25 °C. The initial delay time was set to 60 s, and then additions were performed 19 times with 120 s intervals. The equilibrium dissociation constant (K_d_) was determined using Origin Pro 7 software.

### Analyses of Cytosolic Calcium Dynamics in Guard Cells

For analyses of cytosolic Ca^2+^ dynamics in guard cells, we used *A. thaliana* plants expressing the Yellow Cameleon YC3.6 Ca^2+^ indicator fused to a cytosolic localization signal (NES‐YC3.6).^[^ ^38^ ^]^ Plants were grown on soil under long day conditions (16 h light/8 h dark, 100 µmol m^−2^ s^−1^ of cool white fluorescent lamps) at 22 °C and 75% relative humidity. Guard cells were imaged according to Behera and Kudla.^[^ ^76^ ^]^ Briefly, small pieces of mature leaves of 4‐week‐old plants were glued to a cover slide using Hollister 7730 medical adhesive (Hollister Incorporated, Libertyville, IL, USA); upper cell layers were gently removed with a razor blade, thus isolating strips of the lower epidermis. For the experiments aimed at evaluating the effects of CG and GCG on spontaneous cytosolic Ca^2+^ oscillations the epidermal strips were mounted into an open‐top imaging chamber filled with guard cell closing buffer (5 mm KCl, 10 mm MES, and 50 µm CaCl_2_, pH 6.15 adjusted with Tris‐base),^[^ ^77^ ^]^ and immediately imaged. CG and GCG were added from 2.5 mm stock solutions prepared in the closing buffer to reach a final concentration of 500 µm. As a control, the same volume of closing buffer without catechins was applied. For the experiments where the effects of ABA, ABA + CG, and ABA + GCG were tested, the epidermal strips were kept for 2–3 h in closing buffer under lights (100 µmol m^−2^ s^−1^) to induce stomatal opening, then mounted into an open‐top chamber filled with closing buffer. ABA, ABA + CG, and ABA + GCG were added. ABA was prepared as a 1 mm stock solution in 100% EtOH and was mixed with CG and GCG from 2.5 mm stock solutions prepared in closing buffer to a final concentration of 10 µm for ABA and 500 µm for the catechins (the final EtOH concentration was 1%).

### Wide Field Fluorescence Microscopy

The *Arabidopsis* NES‐YC3.6 indicator line was analyzed with a Nikon Ti‐E inverted fluorescence microscope (Nikon, Tokyo, Japan), using a CFI PLAN APO 20 × VC dry objective. Excitation light at 440 nm (436/20 nm) was produced by a fluorescent lamp (Prior Lumen 200 PRO, Prior Scientific Inc., Rockland, MA, USA) set to 20%. Images were collected with a Hamamatsu Dual CCD camera (ORCA‐D2, Hamamatsu Photonics, Hamamatsu City, Japan). The FRET CFP/YFP optical block A11400‐03 (emission 1: 483/32 nm for CFP and emission 2: 542/27 nm for FRET) with a dichroic 510‐nm mirror (Hamamatsu Photonics) was used for simultaneous CFP and cpVenus acquisitions. Exposure time was 200 ms for guard cells, with 4 × 4 CCD binning. Images were acquired every 5 s for >30 min. Filters and dichroic mirrors were purchased from Chroma Technology. The NIS‐Element (Nikon) was used as a platform to control the microscope, illuminator, camera, and post‐acquisition analyses. Time lapses were analyzed using the FIJI platform.^[^ ^78^ ^]^ Fluorescence intensity was determined over regions of interest (ROI) that corresponded to single guard cells. cpVenus and CFP emissions of the analyzed regions of interest (ROI) were used for the ratio (*R*) calculation (cpVenus/CFP) and plotted against time. cpVenus/CFP ratios were normalized to the initial ratio (*R*_0_) and plotted against time (Δ*R*/*R*_0_). Background subtraction was performed independently for both fluorescence emissions before calculating the ratio. Experiments from at least 40 guard cells were repeated at least *n* ≥ 5. Only Ca ^2+^ oscillations with a peak height that was at least 30% of the highest one observed before treatments were considered true peaks and used for the definition of total peaks’ number. The first 100 s either before or after treatment was not considered for the analysis.

### Measurement of Membrane Potential of Guard Cells

Rosette leaves of *Arabidopsis* were excised from the plants and crushed in a blender. The fragmented epidermis was collected on a nylon mesh and placed in one well of a 12‐well plate with 2 mL of closing buffer. The 12‐well plate was placed in a chamber at 22 °C for 3 h in the light. Ten minutes before the measurement, 2 µm DiBAC_4_(3) (Dojindo Laboratories) and 10 µm ABA, 500 µm CG or GCG were added to the solution where epidermal strips were incubated (final concentration of DMSO was 0.2%), and the fluorescence intensity was measured with a confocal microscope (Leica TCS SP8) using a 40× objective. Images were captured at 488 nm and using 485 to 530 nm long‐pass emission filters with time gating (gate on time: 1.60–5.30 ns) using a white‐light laser. The Leica Application Suite X (LAS X) was used as a software platform. ImageJ as image analysis software was used to separate the raw images into red, green, and blue images, and then the green image was used to calculate the fluorescence intensity of the cells.

### Monitoring of Stomatal Conductance

*Arabidopsis* plants were grown under short‐day conditions (8 h light/16 h dark, 135 µmol m^−2^ s^−1^ with cool white fluorescent lights) in a growth chamber at 23 °C and a humidity of 50–65%. The CO_2_ assimilation rate in intact 3‐4‐week‐old leaves was measured in a measuring chamber with a 6400–02B LED lamp as the light source using a portable gas exchange system (LI‐6400, Li‐COR, Lincoln, NE, USA). The chamber conditions were as follows: CO_2_ and O_2_ partial pressures, 40 Pa and 20 kPa. Vapor pressure deficit ranged from 0.6 to 0.8 kPa, leaf temperature, 23 °C; humidity = 60–75%. When the CO_2_ assimilation rate attained a steady‐state rate at 135 µmol m^−2^ s^−1^, the stomatal conductance was measured. A slit (≈1 mm in width and 1 mm in depth) was made into the petiole with a razor blade and 20 µL of a solution containing 10 µm ABA or 5 µm flg22 (GenScript Japan, Tokyo), and/or 500 µm CG or 500 µm GCG was applied to the slit 5–10 min after transition from the light to the dark. The solution was left to stand for about 2 h, and stomatal conductance was measured. If the solution was depleted before the measurement, an additional 10 µL aliquot was added.

### Chlorophyll Fluorescence Measurements

Chlorophyll fluorescence was measured in intact leaves with a DUAL‐PAM100 (Walz, Effeltrich, Germany) in ventilated room air (40 P_a_ CO_2_, 21 kPa O_2_, at 24 °C) according to Kono et al. 2020.^[^ ^79^ ^]^ Maximum photochemical quantum yield of PSII (*F*_v_/*F*_m_) and effective quantum yield of PSII (Φ_п_) were calculated as (*F*_m_ − F_0_)/*F*_m_ and (*F*_m_′ − F_s_’)/*F*_m_′, respectively. *F*_m_ and *F*_m_′ are the maximum chlorophyll fluorescence with closed PSII centers in the dark and in the light, respectively. *F*_0_ is the minimum level in the dark and Fs’ is the steady‐state level in the light. Non‐photochemical quenching (NPQ) was calculated as (*F*_m_ − *F*_m_′)/*F*_m_′.^[^ ^80^ ^]^

### Statistical Analysis

Tests for differences between two groups were performed using two‐tailed Student's *t*‐test. Multiple‐group comparison was performed by one‐way ANOVA with Tukey–Kramer test or *χ* ^2^ test. Excel (Microsoft), CoStat (CoHort), and Prism 6.01 software (GraphPad) were used for these statistical analyses. *p* values were set as described in the respective figure legends.

## Conflict of Interest

The authors declare no conflict of interest.

## Supporting information

Supporting Information

## Data Availability

Research data are not shared.
