# Supplementary material for: Green Tea Catechins, (−)‐Catechin Gallate, and (−)‐Gallocatechin Gallate are Potent Inhibitors of ABA‐Induced Stomatal Closure
Source: Adv Sci (Weinh). 2022 May 7;9(21):2201403. doi: 10.1002/advs.202201403 (PMC9313475; doi:10.1002/advs.202201403)
Supplement: Supplementary file 1 — Supporting Information [file ADVS-9-2201403-s001.pdf]

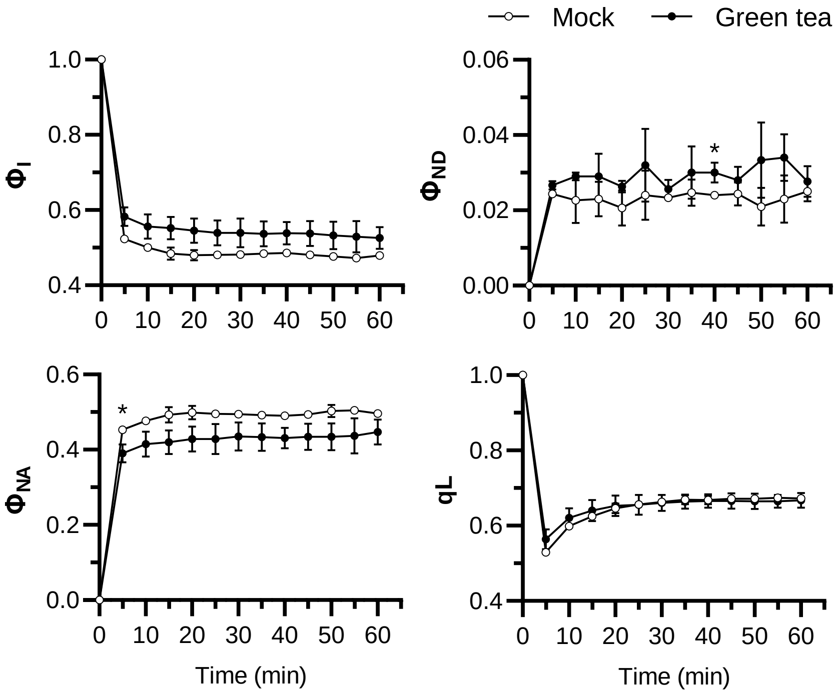

Supplemental Figure 1 Effects of 100% green tea on changes in chlorophyll fluorescence (qL) and redox state of P700 (Φ_I, Φ_{ND}, Φ_{NA}). The measurements were used for the data shown in Fig. 1C. The coefficient of photochemical quenching, qL, a measure of the fraction of open PSII reaction centers, based on the 'lake model' of PSII antenna pigment organization, was calculated as $(F_m' - F')/(F_m' - F_0') \times F_0'/F'$ (81). F_0' is the minimal fluorescence yield in the light and was estimated as $F_0/(F_v/F_m + F_0/F_m')$ (82). $P700^+$ was monitored as the absorption difference between 830 and 875 nm in transmission mode of the Dual-PAM-100. The quantum yields of PSI were determined using the saturation pulse method (83). Φ_I is the quantum yield of PSI photochemistry. Φ_{ND} and Φ_{NA} are the donor- and acceptor-side limitation of PSI, respectively. Data shown corresponds to mean ± SD ($n = 3$). Asterisks indicate significant difference between plants treated with water and that of green tea at different time points (* $p < 0.05$; two-tailed Student's *t*-test).

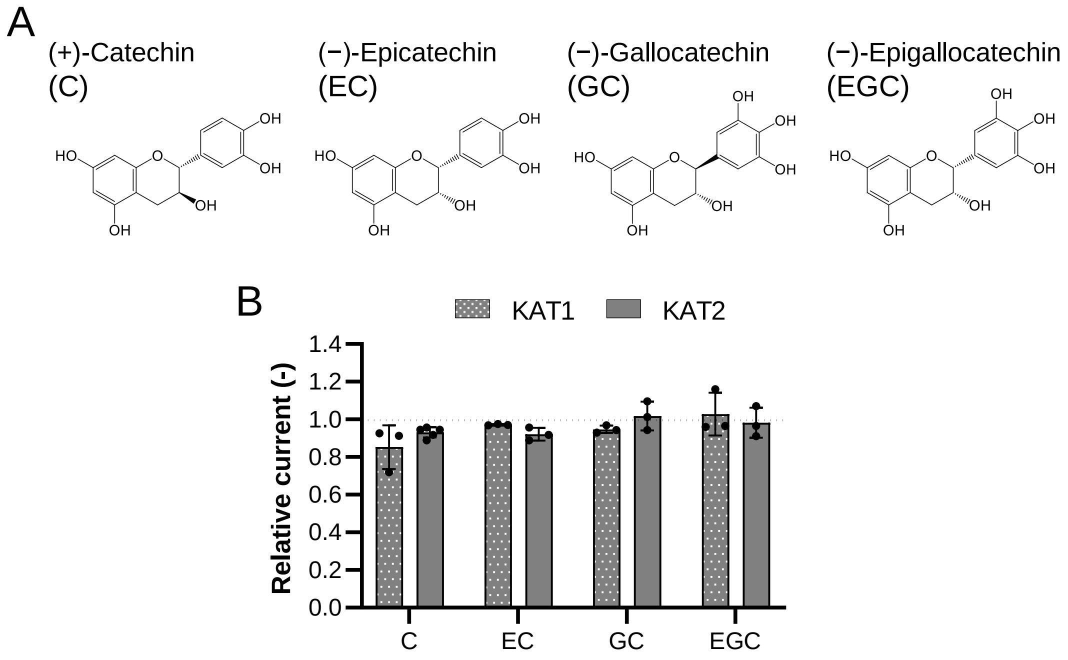

Supplemental Figure 2 Inhibition of whole-cell currents mediated by Arabidopsis K^+ channels by catechins without galloyl ester.

(A) The chemical structures of the four catechins. (B) Effect of four catechins on KAT1 and KAT2. Two-electrode voltage clamp was carried out for each channel with or without 500 µM tetraethylammonium (TEA) or catechins in the external solution respectively, and relative currents were plotted (n = 3-5, mean ± SD). The current value was collected at the end of the pulse −170 mV for KAT1 and KAT2.

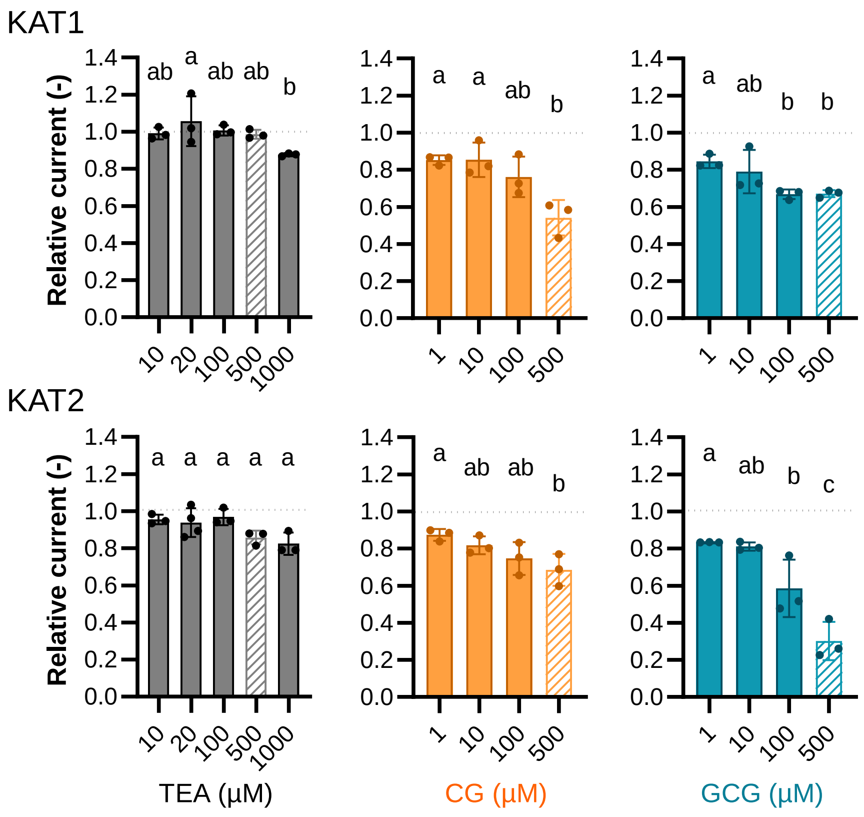

Supplemental Figure 3 Inhibition properties of CG, GCG and TEA on *Shaker* K^+ channels.

Relative currents of KAT1 and KAT2 were plotted against TEA, CG or GCG concentrations (1, 10, 20, 100, 500 or 1000 µM, mean ± SD). The relative current was determined by calculating the values obtained at the end of 0.5 s pulses at −170 mV for KAT1 and KAT2 in a solution containing TEA, CG or GCG as a percentage of currents without addition of TEA, CG or GCG. The data of KAT1 and KAT2 with 500 µM TEA, CG or GCG are same data of Figure 3 (B). Each data point corresponds to mean ± SD (n = 3-4). Bars marked with different letters are significantly different ($p < 0.05$) by one-way ANOVA with Tukey-Kramer test.

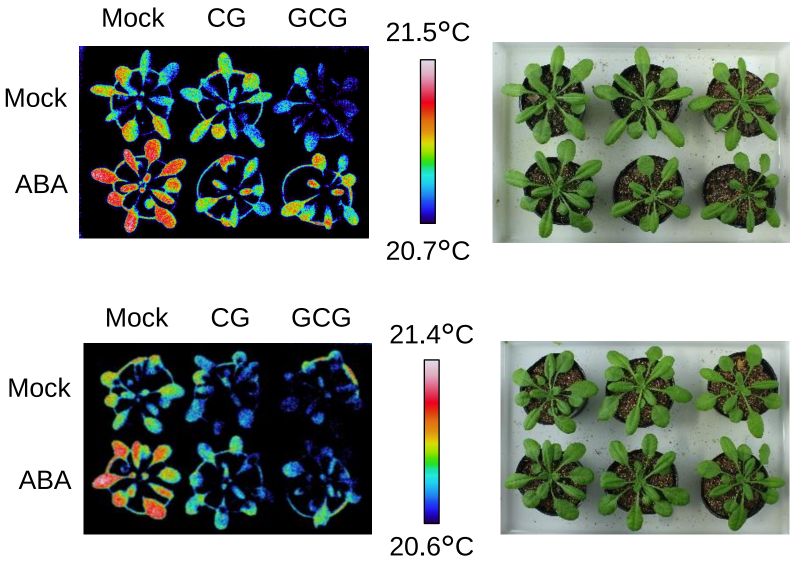

Supplemental Figure 4 Additional data of false-color infrared image of plants treated with CG or GCG in Figure 7B.

The 3-4-week-old plants were sprayed with 500 μM CG or 500 μM GCG without or with 10 μM ABA. The infrared thermography was taken 3 h after the treatment.
